# Light People: Prof. Henry Snaith’s (FRS) perovskite optoelectronics journey

**DOI:** 10.1038/s41377-024-01668-y

**Published:** 2025-01-01

**Authors:** Ruidong Xia, Ying Hu

**Affiliations:** 1 Key Laboratory for Organic Electronics & Information Displays (KLOEID), Jiangsu-Singapore Joint Research Center for Organic/Bio Electronics & Information Displays, Institute of Advanced Materials (IAM), Nanjing University of Posts and Telecommunications, 9 Wenyuan Road, Nanjing, 210023 China; 2 Executive Management College of CHN ENERGY, No.7 Binhe Avenue, North District of Future Science City, Changping District, Beijing, 102211 China; 3 Key Laboratory of Terahertz Optoelectronics, Ministry of Education, Capital Normal University, 105, West Third Ring Road, Haidian District, Beijing, 100089 China

**Keywords:** Optics and photonics, Organic LEDs, Electronics, photonics and device physics, Optical materials and structures

## Abstract

In 2012, Prof. Henry Snaith demonstrated the first solid-state perovskite solar cell (PSC) with an efficiency of 10.9%, igniting a surge of interest and research into perovskite materials for their potential to revolutionize the photovoltaic (PV) industry. Over the past two decades, perovskite optoelectronics have made remarkable progress, with significant improvements in efficiency, stability, and commercial viability, which has transformed these materials from a scientific curiosity into a leading platform for a wide range of applications, particularly in PVs and light-emitting diodes (LEDs). Prof. Henry Snaith’s election as a Fellow of the Royal Society (FRS) credits to his groundbreaking discovery of the use of perovskites in efficient solar cells. In addition to his academic role, Henry co-founded and served as the Chief Scientific Officer (CSO) of two spin-off companies, Oxford PV Ltd. and Helio Display Materials Ltd., which focus on commercializing metal halide perovskite PVs and light-emitting applications, respectively. His team has led the global R&D community in advancing the fundamental understanding and practical use of perovskites since 2012. On 5th September 2024, Oxford PV announced the world’s first commercial sale of next-generation perovskite tandem solar panels, which generate up to 20% more energy than a standard silicon panel. In an insightful conversation with *Light: Science & Applications*, Prof. Henry Snaith, a pioneer of metal halide perovskite optoelectronics, shared his story on how scientific curiosity, close observation to unexpected results, and serendipity led to the discovery of perovskite as a solid light absorber, as well as the key findings and breakthroughs to achieve the remarkable efficiency of PSCs. He highlighted the significant contribution of his team to the progress of PSC technology from its initial discovery to its current exciting commercialization status; this includes the development of tandem solar cells and the exploration of p-i-n configurations for better stability. Moreover, he expressed his views on the future of perovskite LEDs and environmental and safety concerns related to perovskite optoelectronics technology. The interviews further explored Henry’s journey from an undergraduate physics student to a renowned scientist. His career success is undoubtedly driven by his ambition for immediate real-world impact and his relentless pursuit of more efficient, low-cost, and sustainable energy solutions to address global environmental challenges. When asked about the potential for a Nobel Prize, Henry acknowledged that PSC technology could be worthy of such recognition, given its scientific advancements and significant contributions to addressing the global challenge of climate change. Looking ahead, Henry has expressed an interest in contributing to public policy, particularly in the areas of renewable energy and education reform, with an emphasis on the creation of an inclusive education system that better supports neurodiversity.

**Figure:**
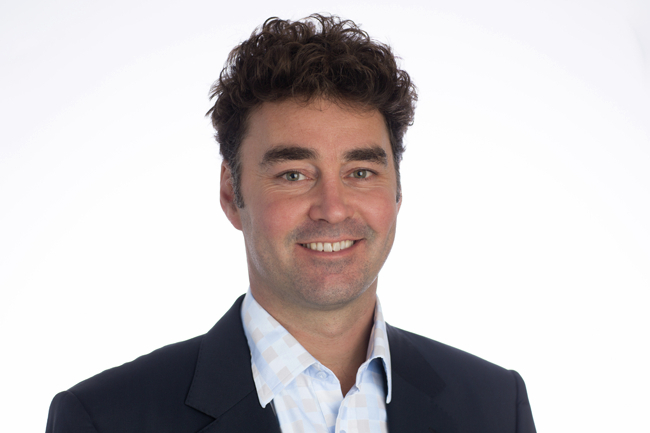
Prof. Henry Snaith

**Biography:** Prof. Henry James Snaith was born in 1978. He was educated at the independent Gresham School in Norfolk from 1989 to 1996. He completed his undergraduate studies in 2001 at the University of Bristol and PhD in 2005 at the University of Cambridge on polymer solar cells supervised by Prof. Sir Richard Friend. After finishing his PhD, Henry conducted two years of postdoctoral research with Prof. Michael Gratzel at the Ecole Polytechnique Federale de Lausanne (EPFL). He then returned to the Cavendish Laboratory as a Junior Research Fellow at Clare College, Cambridge University, in 2006. Following this, he was appointed a Research Councils UK (RCUK) research fellow in 2007 and then promoted to reader and professor in physics at the Clarendon Laboratory at the University of Oxford. Now, he is the Binks Professor of Renewable Energy. Henry’s research has focused on new materials and device architectures for the future generation of low-cost photovoltaics. His research group works with organic, metal oxide, and metal halide perovskite semiconductors processed via solution- or vapor-phase deposition methods. His interdisciplinary work ranges from new material synthesis and discovery to device fabrication and development, advanced characterization methodologies, and theoretical modeling. Henry’s achievements include the discovery of high-efficiency solid-state organic-inorganic metal trihalide perovskite-based thin films and mesoporous solar cells. He co-founded Oxford PVs in 2010, initially to commercialize solar cells for building integrated utility-scale photovoltaic applications. He is also the co-founder and CSO of Helio Display Materials Ltd. to commercialize metal halide perovskites for light-emitting applications. Helio Display Materials is presently focused on perovskites for color conversion, enabling full-color micro-LED displays for augmented reality applications. He was awarded the Institute of Physics (IOP) Clifford Paterson Medal and Prize in 2012 and the MRS Outstanding Young Investigator Award in 2014. He was elected a Fellow of the Royal Society in 2015. In May 2016, he was awarded the EU-40 Materials Prize from the European Material Research Society. In October 2017, he was awarded the IOP James Joule Medal and Prize. In September 2020, he was awarded the Becquerel Prize. He won the 2021 Leigh Ann Conn Prize for Renewable Energy from the University of Louisville and the 2021 Rank Prize in Optoelectronics. He was also named one of Nature’s ten people who mattered in 2013 and ranked number two on the list of *The World’s Most Influential Scientific Minds*, a citation analysis identifying the scientists who have made the most significant impact within their respective fields of study by the Intellectual Property (IP) and Science business of Thomson Reuters in 2016.

**Q1. In 2012, You demonstrated the first solid perovskite solar cell (PSC) with an efficiency of 10.9%, which led to a spurt in PSC research. Why did you choose perovskite for solid dye-sensitized solar cells? What triggered your approach to device fabrication, such as replacing TiO**_**2**_ **with the insulator Al**_**2**_**O**_**3**_**, using mixed-halide CH**_**3**_**NH**_**3**_**PbI**_**3-x**_**Cl**_**x**_**?**

After I began my faculty position at Oxford in 2007, I had a Japanese–UK collaborative grant with a very good colleague and friend of mine, Takurou Murakami, who was a lecturer at Toin University at the time. I sent a PhD student to Toin University, and we were discussing and trying to work out what he should work on. We decided that the student should explore perovskite materials as absorbers in our solid-state dye-sensitized solar cells (DSSCs). At the time, I still believed that a porous TiO_2_ structure was ideal for solid-state dye cells, but we needed more effective light absorbers. The dyes we were using didn’t absorb strongly enough, so we tried to infiltrate the pores with light-absorbing polymers like P3HT and other semiconducting polymers. Unfortunately, we couldn’t achieve high current densities with these polymers. We also investigated quantum dots, and I personally synthesized lead sulfide quantum dots in the lab, but we struggled to get a high color ratio, resulting in only a few percent efficiency. During our search for new light absorbers, Tsutomu Miyasaka from Toin University published a study on using perovskites on porous TiO_2_ in a liquid electrolyte dye cell in 2009. I thought we could try these materials in our solid-state dye cells, but at the time, I had no idea how well they would perform. I expected perhaps 1 or 2% efficiency. Surprisingly, our first batch of solar cells using perovskite absorbers with TiO_2_ already achieved over 6% efficiency—it worked straightaway.

As a physicist, I wanted to understand the physics behind this 6% efficiency, so we conducted extensive characterization of our devices. One key measurement was charge extraction. We pulsed the devices with light and used an oscilloscope to measure how long it took for the charge to rise and then flow out of the device. We observed that in the perovskite-sensitized devices, the current decay was much faster than that in the dye-sensitized devices, suggesting faster charge extraction with the perovskite; this made me wonder if the perovskites were not only absorbing light but also facilitating electron transport. To test this, we replaced the porous TiO_2_ with a porous insulator in some test cells to see if charge transport occurred solely through the perovskite. These test cells weren’t intended to be efficient solar cells, but rather a reference to measure charge transport. In our first batches, we made 32 cells with TiO_2_ and two with insulator Al_2_O_3_ as the reference. When we measured them using a solar simulator, the best efficiency from the TiO_2_ cells was 7.2%, but the two Al_2_O_3_ cells surprisingly reached 10% efficiency. The open-circuit voltage (Voc) increased dramatically from ~0.8 V to 1.1 V. This massive jump in Voc was completely unexpected. After that, we looked at other insulators like silicon oxide and zirconium oxide, which had similar porosity and structure to the TiO_2_, but were non-conductive. They were all similar, but Al_2_O_3_ remained the easiest to process. This was a surprise and totally unanticipated result. This discovery was crucial because it demonstrated that the perovskite itself could function effectively as both the light absorber and the charge carrier transporter, reducing the reliance on a separate electron-transporting layer.

Another breakthrough occurred when we used a mixture of chloride and iodide, specifically CH_3_NH_3_PbI_3-x_Cl_x_, which was another somewhat serendipitous discovery. One of my students at the time was highly skilled in combinatorial exploration, and he experimented with various combinations of lead and tin salts—mixing iodide, bromide, and chloride with methylammonium iodide, methylammonium bromide, and methylammonium chloride. He explored all possible compositions to observe their impact on the film properties. One reason for this comprehensive comparison was our lack of understanding of perovskite chemistry at that stage. We didn’t know the extent of halide mixtures that could form. We wanted to determine whether iodide could form an alloy with chloride or if only a small amount of chloride could be incorporated into the iodide. It turned out that only a small amount of chloride was mixed with the iodide, leading to two significant effects. First, we achieved defect passivation, resulting in a well-passivated and highly emissive perovskite. This unexpected outcome laid the foundation for many subsequent discoveries. Additionally, our process—using lead chloride and methylammonium iodide to form the 3D perovskite—required adding a substantial amount of methylammonium iodide in excess. This approach led to what we now refer to as a precursor phase route. When the film was cast, it didn’t immediately form perovskite. Instead, a lower-dimensional compound was initially formed, which was then annealed into perovskite. This method produced large crystalline domains with highly crystalline material.

When we compared our material processed from lead chloride and excess methylammonium iodide to the material processed by Tsutomu Miyasaka, who used a stoichiometric mixture of methylammonium iodide and lead iodide in a solvent, we found significant differences. Miyasaka’s material had tiny, nanocrystalline domains and was full of defects, making it poorly emissive and not work at all on the Al_2_O_3_. In contrast, our material was highly crystalline and passivated, allowing for excellent long-range conduction and a high carrier lifetime. This development enabled us to move away from Al_2_O_3_ and rely solely on thin-film perovskite, which transports both electrons and holes.

In summary, our discoveries were the result of a combination of coincidences and serendipitous effects. If we had been experts in perovskites at the time, we would never have tried to make the material the way we did because it didn’t initially make sense. It only made sense once we realized that a small chlorine content could passivate defects and improve stability and performance and that the precursor phase process allowed the grains to grow into large, crystalline domains. We didn’t plan for these outcomes, but when they happened, we recognized their transformative potential. We realized that perovskites weren’t just light-absorbing quantum dots—they were proper semiconductors with properties similar to gallium arsenide in terms of photoluminescence efficiency and carrier lifetime. This material, which we just spin-coated from this recipe, turned out to be a game changer.

**Q2 Since that success, you have published over 100 papers and started companies in perovskite PVs. Would you please highlight the major progress/milestones in PSC research you did in last 20 years?**

The first use of perovskite materials in DSSCs in 2009 showed efficiencies of just a few percent, but this work laid the foundation for future research. Since we reported our findings, nearly everyone working on organic PVs shifted their focus to the planar heterojunction structure. PSC efficiencies rapidly increased, surpassing 20% within a few years. The first efficient perovskite LEDs were reported around 2014.

During the two decades, another significant discovery we made, likely in 2017, involved the device configuration. Initially, we used what we called an n-i-p structure, where the n-type material, typically TiO_2_, was at the bottom, and the p-type material, an organic hole conductor, was on top. To explore other possibilities, we also created p-i-n cells, where the p-type material was placed at the bottom, and the n-type material was on top. The first materials we used in the p-i-n cells were PEDOT:PSS and PCBM fullerene, which were familiar with organic PVs. This structure worked reasonably well, though it never matched the performance of the n-i-p structure. However, in 2017, we discovered that the p-i-n structure was significantly more stable than the n-i-p configuration. As a result, we shifted ~90% of my research group’s efforts to p-i-n cells. This configuration is also highly compatible with multijunction cells, which I’ll discuss shortly.

Beyond stability, another critical factor is efficiency, especially in the context of existing silicon technology. The industry has made significant advances, and while perovskites have now reached the efficiency levels of silicon in single-layer configurations, it’s challenging to introduce a new technology that merely matches silicon’s efficiency. To truly compete, we need to offer much higher efficiency. Recognizing this early on, we shifted our focus to tandem solar cells. In fact, our first patents, submitted before we published our initial paper, identified the high voltage of perovskites as particularly suitable for tandem solar cells. These patents included claims about using perovskites in tandem solar cells, and we have continued working on this concept ever since.

One of the major challenges in this area has been tuning the band gap of perovskites, where mixed halides have proven to be very relevant. By adjusting the ratio of iodide and bromide, we can achieve the ideal band gap for pairing with silicon. Additionally, by transitioning from lead to tin, we can lower the band gap of perovskites, enabling the creation of all-perovskite tandem or triple-junction cells using thin-film materials with precisely tuned band gaps. Starting in 2014, a significant portion of our research has focused on optimizing these materials and perfecting device stacks for multijunction cells.

In the past decade, other major developments have also emerged. Stability has become an increasingly important theme, particularly in understanding how to slow down photo-induced degradation, which occurs due to temperature, light, and electric fields. Early on, there were many questions about the role of ions in perovskites and whether our reported efficiencies were accurate. Initially, we reported current-voltage scans, but we soon realized the need to report steady-state efficiency. As a result, we introduced steady-state measurements for perovskites, as well as maximum power point tracking. Today, all record efficiencies are reported as maximum power point tracked or steady-state efficiencies.

While we now have a better understanding of the effect of ions on efficiency, the impact of mobile ions on long-term operational stability remains a hot topic of research. When these materials are exposed to high temperatures, sunlight, and varying environmental conditions, more mobile ions can be generated in the perovskites. These ions can affect the electronic properties and overall performance of the devices. Although we’ve gained considerable understanding in this area, there is still much more work that can be done.

Q3. Soon after demonstrating the high-efficiency perovskite PVs, you started a company, Oxford PV, to work on commercial perovskite PV products. At that time, you believed the PSC would be commercially available in the next decade. So, now how far we are to see the real PSC panel on the market? * As you said, the stability of perovskites is a big challenge. So, what, you think, could the lifetime of commercial PSCs be?

(with excitement) Within this quarter, there will be significant development. The utility-scale solar panels are progressing well. These panels consist of 72 cells, with each panel measuring approximately two by one meter. They are intended for a solar farm, so they’re going to a real solar farm customer. This is proper commercial shipping, and payments have been made. I can’t disclose the cost at this point. There will likely be a press release from Oxford PV soon, maybe in the next week or a few days, but definitely within this quarter, Q3. These panels use tandem perovskite on silicon technology, which is very efficient. In the lab, efficiency has exceeded 33%, but for real-world modules, the certified efficiency is ~26.9%. The first product we are selling has a whole-area efficiency of ~24.5%. Regarding the product’s lifetime, I can’t disclose the exact predicted lifetime, but it’s acceptable to our utility customers. The target is to warrant the panels for 25 years once the full production line is ramped up and we’re producing at high volumes. Following this initial deployment, production will ramp up significantly. The company is currently increasing production and has a production line in Brandenburg, Germany. However, this line will max out at 100 megawatts, so we are raising funds to build a gigawatt-scale production line.

**Figure:**
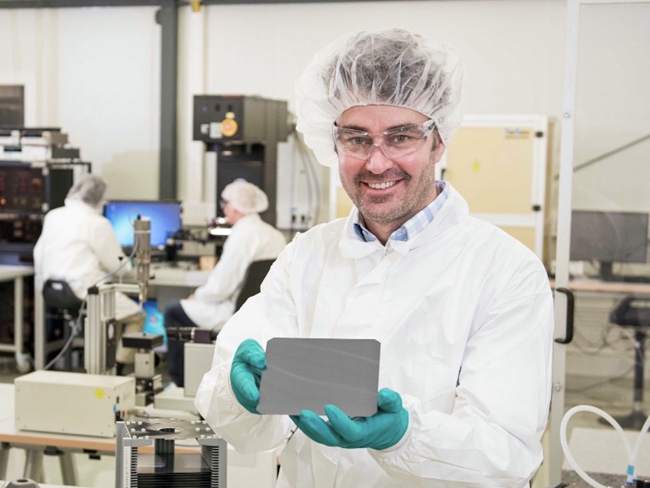
Prof. Henry Snaith holds a perovskite tandem solar cell in the laboratory

*On 5th September 2024 (2 weeks after the interview), Oxford PV announced the world’s first commercial perovskite tandem solar panels that produce 20% more energy than a standard silicon panel. The panels are powered by perovskite-on-silicon cells produced at Oxford PV’s megawatt-scale production line in Brandenburg an der Havel, Germany.

**Q4. It is really a very exciting news! Congratulations! Does this mean governments allow the PSC to be widely used? One negative aspect of perovskites is the fact that lead has been a major constituent of all highly performing PSCs to date, raising toxicity issues during device fabrication, deployment, and disposal. What is your opinion on this issue?**

The issue of lead toxicity in PSCs does need to be considered, especially given the growing emphasis on environmental sustainability and public health. Our PSC modules are fully compliant with all the necessary regulations concerning the materials used. They meet existing environmental and safety standards. Although there is a very small amount of lead in the perovskite layers, it’s important to note that these layers are extremely thin, making the absolute amount of lead in the solar cells very low. Additionally, the modules are laminated, which means that any lead in the solar cells will not leach out, and if the modules are recycled at the end of their life, the process is straightforward. The lead and other valuable materials, such as indium, can be easily recovered. Tests have shown that even if the modules are ground up, the lead does not leach out because it is securely held within the laminated material. Customers don’t need to worry about any negative environmental impact.

As for manufacturing, any potential industrial waste must be carefully captured and treated appropriately. It’s crucial to ensure that the production process is safe, not just to prevent contamination of the product, but also to protect workers. In fact, people are not in direct contact with these materials during production since the materials are deposited in a controlled environment separate from human operators. There’s no need for concern as long as the factory is designed with human health in mind, and emissions are properly managed to prevent the release of toxic materials. While lead is a concern for many, it’s actually one of the less problematic materials in this context due to its minimal quantity. The most hazardous material used in the production of perovskite-on-silicon tandem cells is actually silane, which is used for the growth of amorphous silicon in heterojunction cells. Silane requires significant safety measures, but lead is relatively straightforward to handle.

In terms of sustainability, it’s essential to consider the full lifecycle of the module, including the sourcing of raw materials, the energy required for production, and the management of wastewater and emissions. However, lead contributes <1% to the overall ecotoxicity in comprehensive lifecycle assessments. Therefore, governments and policymakers should not be overly concerned about the lead content. Instead, they should focus on ensuring the safe and responsible use of lead within the industry, as the risks are minimal when properly managed.

**Q5. Given that the PSCs have already commercially available, what, do you think, the academic research should be focused on in perovskites optoelectronics?**

This is just the start of the journey for perovskites. With multijunction cells and even more advanced concepts, there is the potential to keep increasing the efficiency of the solar cells towards and beyond 50%. This will take a lot more fundamental work from academics. Degradation and long-term stability remain poorly understood, so there is still a lot of benefit in investigating and learning more about degradations that can occur in metal halide perovskites and devices. For solar cells, similarly to what is happening with silicon modules, we will need to keep extending the operational lifetime to 50 years. LEDs are far less developed for perovskites, there we need to increase the efficiency of the pure-blue LEDs, which have an emission wavelength of ~470 nm, and increase the operational stability by two to three orders of magnitude as well. There is also a big opportunity for finding other uses for this highly intriguing family of semiconductors.

**Q6. The perovskite LEDs and lasers have also been demonstrated soon after high-efficiency perovskite PVs was achieved. What are the major advantages the perovskite LED over its counterpart, such as flexible and solution processable organic LED and very low-cost GaN LED?**

One of the significant advantages of perovskites over organic materials is their superior color purity and their ability to reabsorb and reemit light within the active layer, a process known as photon recycling. This property allows perovskites to achieve much higher radiative efficiency out of a device, as compared to organic materials.

Materials like gallium nitride (GaN) LEDs can achieve very high quantum efficiencies, often exceeding 80%. This high efficiency is due in part to the minimal losses in emitted light. In contrast, organic light-emitting diodes (OLEDs) are typically limited to ~30–35% quantum efficiency. The primary reason for this limitation is the significant Stokes shift in organic materials, which causes a large portion of waveguided light to be absorbed parasitically rather than being reabsorbed and reemitted by the emissive layer.

Perovskites, on the other hand, have a much smaller Stokes shift. This means that the waveguided light in perovskite materials is more likely to be reabsorbed by the emissive layer and then reemitted. This photon recycling reduces parasitic absorption and leads to higher overall efficiency in light emission. As a result, perovskite-based devices have the potential to achieve higher efficiencies than those based on organic materials.

In the context of LEDs, while GaN-based blue LEDs are very efficient, the red and green LEDs are typically less so. This is particularly problematic in applications like micro-LED displays, where efficiency losses become more pronounced as the pixel size decreases to just a few microns. Our startup company, Helio Display Materials, is mainly focused on color conversion for micro-LEDs. We are addressing this challenge by using perovskite nanoparticles or quantum dots as color converters. In this setup, blue GaN LEDs provide the light, which is then absorbed by the perovskite and reemitted in different colors.

By using blue GaN LEDs for the primary light source and perovskites for color conversion, we can achieve higher overall efficiency and brightness in micro-LED displays. This approach helps to overcome the efficiency limitations of red and green GaN LEDs, making perovskite-based color converters a promising solution for next-generation display technologies.

**Q7. Do you think once you did the solar cell, it’s easier to use a similar approach to fabricate perovskite LEDs. What were the different research focus when you worked on the perovskite LEDs?**

Despite the apparent similarities between PSCs and perovskite LEDs, the transition from developing efficient solar cells to creating efficient LEDs is not as straightforward as one might expect. While both technologies rely on similar materials, the specific requirements for LEDs introduce new challenges that necessitate a different approach and expertize.

In our group’s research experience, making efficient LEDs from perovskites involved overcoming various hurdles that are distinct from those in solar cell fabrication. For instance, the contact layers, the smoothness, and the integrity of the layers are crucial factors that differ between the two types of devices. This means that even a team well-versed in solar cell development cannot immediately apply the same knowledge and techniques to create efficient LEDs. It requires a focused effort to adapt the patterns, structures, and processes specific to LEDs.

However, the situation could be different when using solution-processed layers with evaporated layers. The tools we have, such as the large cluster tool in Oxford, can evaporate both perovskites and organics. Once high-efficiency LED processes are established, they could be more easily reproducible. This is because the processes would rely more on established recipes rather than the skill of individual researchers, making it easier for new researchers to produce efficient and stable LEDs consistently.

Regarding tandem perovskite LEDs, the idea of using multijunction designs to improve LED performance is promising. The main challenge with perovskite LEDs today is their long-term stability, especially under an electric field, where they degrade relatively quickly compared to OLEDs. Multijunction LEDs could address this by requiring less driving voltage to achieve the same brightness, potentially enhancing their stability and efficiency.

Our work on tandem solar cells, particularly with up to four junctions, provides a solid foundation for exploring multijunction LEDs. The idea of using this approach to achieve lower driving voltages and higher brightness in LEDs is an exciting direction for our research. It could lead to significant advancements in LED technology, provided the stability issues can be adequately addressed.

**Q8. You are renowned scientist in energy research now. Talking about your personal career journey, when did you start to be interested in solar cell and why did you decide to do organic photovoltaics (OPV) research for your PhD in Cambridge? What benefit you got from your PhD for your later successful career?**

I developed a strong interest in science during my undergraduate studies in physics. I knew I wanted to go into research, but more importantly, I wanted to work on something useful—something that could make a tangible impact. Renewable energy felt like a field where I could contribute meaningfully, so I started exploring career options in wind energy, nuclear fusion, and PVs. Wind energy seemed too focused on engineering, and nuclear fusion, while promising, appeared unlikely to be realized in my lifetime. PVs, on the other hand, seemed like an area where I could make a difference relatively soon. It was a field where I could apply myself and contribute to something with immediate real-world impact.

When I received my MSci, I initially wanted to work in industry because I was more interested in creating practical solutions that could affect the world than in purely academic research. I applied for several jobs, and I received a reply from BP Solar. They told me that to work in their R&D department, I would need a PhD. That response made it clear that I needed to pursue further studies. As I began looking for PhD programs in sustainable energy, I decided not to focus on silicon PVs because it was already industrialized, and I thought there might not be many exciting academic research opportunities left in that area. Then I saw an advertisement for a PhD position with Richard Friend at Cambridge University to do organic PVs research, which sounded interesting and innovative. That’s what led me to choose Cambridge for my PhD in 2001.

My PhD supervisor, Richard Friend, was already a prominent figure in organic optoelectronics, though I didn’t know how well established he was when I started my PhD. It turned out to be a fortuitous decision, as his influence and connections greatly enriched my experience. As an undergraduate, I was surprised to discover how directly relevant academic research can be to industry. Richard Friend’s involvement with Cambridge Display Technologies (CDT) and Plastic Logic exposed me to the practical applications of research. My interactions with scientists at CDT helped me understand that academic research often intersects with industry through patents and collaborations with startups or established companies. This realization significantly increased my enthusiasm for academic research.

I grew in confidence and developed research skills during my PhD, which have been important for my later career. When I started my PhD, I was unsure of my potential in academia. However, after three years, I found that I was reasonably proficient as a scientist and decided to continue in the field. My knowledge and experience in organic semiconductors, polymers, and small molecules also proved valuable during my postdoctoral studies on solid-state DSSCs.

**Q9. Obviously, you had a very successful career in PSC research, while your PhD research was on OPV, a very different field. Why did you shift your research focus from polymer PVs to DSSCs? What made you decide to do postdoc in Michael Gratzel’s group at the Ecole Polytechnique Federale of Lausanne (EPFL)?**

After completing my PhD, I was eager to continue my research in solar cells but was also very interested in branching out in a slightly different direction. I found the DSSC, particularly the solid-state variant, to be a promising area. One of the challenges with organic solar cells is controlling their microstructure, which I spent my entire PhD working on. In contrast, DSSCs allow for a fixed microstructure in the metal oxide, which can offer more stability. Organic solar cells often face issues with domain growth and nanostructure coarsening, which can hinder the generation of free carriers. The porous anode in dye-sensitized cells is structurally stable and less prone to such issues, making it a potentially more robust platform for long-term stability.

I was also looking for a nice exciting place to live. I love skiing. EPFL in Lausanne, Switzerland, was a good choice. EPFL was renowned for its well-established group working on DSSCs, and it closes to the mountains. So, these are two reasons I applied and moved to EPFL in Lausanne. The primary challenge with dye-sensitized cells was that efficient devices typically used liquid electrolytes. My goal at EPFL was to work on solid-state DSSCs using organic hole conductors, aiming to address this issue and advance the technology.

**Figure:**
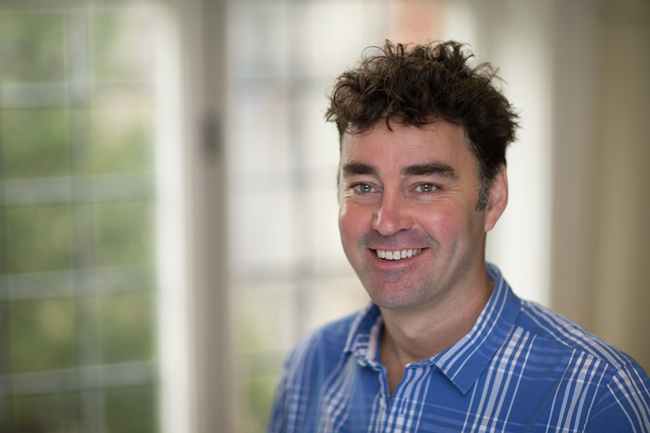
Prof. Henry Snaith as an early career researcher

**Q10. Based on your journey toward PSC research, what is your advice to researchers (young scientists)?**

It was a mix of serendipity and keen observation that led to our breakthrough. We initially didn’t set out to discover that removing the conductor would make the solar cells significantly better. Our goal was simply to understand the transport mechanisms within the perovskite material. The unexpected improvement we observed when we removed the conductor was a complete surprise. With hindsight, we now understand exactly why it led to better performance, but at the time, it was a startling revelation. What I believe made a difference was our meticulous approach to observing and interpreting our results. The most intelligent or best scientists in the world would probably not have planned to go through the set of experiments that we did. I think most other people would have overlooked the faster charge extraction in the “perovskite sensitized” cells. It was just closely observing what we’ve got and then recognizing what it means.

During that period, there was a significant debate in the community about whether to use mesostructures or planar heterojunctions for PSCs. For ten years of my career, including the years at Oxford, I had focused on developing nanostructured and mesoporous materials, including efforts to create ideal mesoporous TiO_2_ films and even large single-crystal porous TiO_2_ domains. I thought this was the ideal porous n-type semiconductor. So at the time, my whole career had been focused on meso- and nanostructures. Then, we find this perovskite material can be processed really simply, and it just works as a thin film. It was quite a surprise and a shock that we no longer needed any of the mesoporosity stuff. Everything, all the work I’d done previously and quite a lot going on in the group at the time was no longer needed or relevant. We had to manage a radical shift from everything I had previously worked on when we encountered perovskite. This shift required us to quickly adapt and focus solely on solid, thin-film perovskite technologies. The transition away from mesoporous structures was surprising and disruptive, especially given the previous decade of emphasis on such structures.

The research community took about ten years to fully embrace this shift. Despite ongoing efforts by some groups to refine porous TiO_2_ structures, it became clear that planar heterojunctions were more effective. Indeed, this year, the world record efficiency for a PSC is held by a planar heterojunction design, confirming the advantage of solid, flat layers over mesoporous structures.

Looking back to my research career, I would like to say this journey was marked by a willingness to experiment with unconventional approaches and to question existing paradigms. The rapid progress we made was partly due to the collaborative and open environment in the research community, where ideas could be exchanged freely and tested quickly.

**Q11. Going back to your early-stage education, you were educated at Gresham’s School, a famous independent school where Sir James Dyson, a well-known UK inventor and entrepreneur, studied in 1956–1965. Why did your parents choose this school and be willing to pay the expensive fee for your high school education?**

My early years in school were challenging. I was pretty bad at school when I was very young. I was not naughty, just didn’t concentrate very much and didn’t really find school very interesting. I was more interested in playing football and running around than in my lessons, which meant I was at the bottom of the class. However, things began to change around the age of nine when we started having proper science lessons. I found science fascinating, which sparked my interest in school and helped me improve academically.

My parents played a crucial role in this transformation. They prioritized our education. Initially, I attended a local state school, but the quality of education was lacking. Recognizing the need for a better learning environment, my parents decided to invest in private education for me and my sister, although they didn’t have a lot of money—running a hotel and restaurant in Norfolk. So, their support and investment in my education were instrumental in my academic development. I began attending private school around the age of seven. One of the best schools in the county was Gresham School, where I studied from the age of 11 until the end of my sixth form.

**Q12. Many people (and I) believe that you will be one of the Nobel Prize winners for your work on PSCs. How do you think? If so, whom do you think you should acknowledge?**

The development of PSCs is not only a scientific achievement but also a step forward in our collective efforts to address the global challenge of climate change. I believe that perovskites, as a technology, could indeed be worthy of a Nobel Prize. It would be appropriate if such recognition came once the technology was fully proven and its impact had been demonstrated. If that happens, I would be honored to be among those recognized for their contributions. However, I do not want to preempt any outcomes, and I acknowledge that many others have made significant contributions to the field of perovskites as well.

If a Nobel Prize were awarded in this area, several people come to mind who would deserve recognition. Firstly, Tsutomu Miyasaka’s group published the first paper on perovskites, which was foundational for the field. Without his pioneering work, we might not have explored perovskite materials or developed the technology we have. So if I were to get it, I think he should as well. Additionally, my past supervisors—Richard Friend and Michael Gratzel—played crucial roles in guiding and supporting my work. Of course, I must also mention my parents, whose unwavering support throughout my education has been instrumental in my journey. Researchers in my group have been key to our success. The work on PSCs has been a team effort, and I am fortunate to have worked with such talented and dedicated researchers.

**Q13. As a renowned scientist, you are still very young. Many well-known scientists transition into management roles after achieving recognition in their fields. Besides conducting research and running your perovskite commercial application related companies, have you considered to take management roles as well?**

I don’t know the thing about the future. However, I wish I could do something in two areas. Looking ahead, the next decade is crucial for our planet. We must accelerate the transition to renewable energy to address climate change effectively. I am committed to contributing to this transition in any way I can. Public engagement and influencing policymakers are essential. I do not know if I would like to be MP. However, I could certainly try to get our voices heard and influence the public, decision-makers, and politicians into making the right decisions about how we transition to a clean, carbon-free society.

Regarding public education, I believe it would be beneficial to improve the state education system. Access to high-quality education should not depend on private schooling. As someone who may be dyslexic, I’ve seen firsthand how the state education system can fall short in accommodating special educational needs. This is a significant issue that needs addressing in the UK. We need a system that supports cognitive diversity and neurodiversity better to foster a more inclusive and effective learning environment. I would like to influence the decision-makers towards effective policies to ensure that all students, regardless of their learning differences, receive a quality education.

**Q14. I have noticed that some media reports with your photo have such descriptions as “a science star who is both smart and handsome” and “scientists who love to laugh are not too bad luck”. Do you think you are naturally optimistic? How would you be able to get over the hill eventually at any dark time in your research life? How could you balance your busy life between academic research, R&D innovation, and family life? In your opinion, how do researchers maintain a happy mood under intense research work?**

In general, science is a journey filled with challenges, setbacks, and moments of doubt. However, it’s the potential to discover something new, to contribute to the field that keeps me—and many others—moving forward. I would say that optimism is a crucial trait for anyone engaged in research. Optimism means maintaining perspective, finding joy in the process rather than just the outcomes, and knowing that every setback is an opportunity to learn and grow. During dark times in research, it’s important to remind oneself why you started in the first place. I’ve always been motivated by a deep curiosity and a desire to solve problems that could have a real-world impact.

Now, I have a large research group and two R&D companies. Work inevitably comes with its stresses. Technologically, we’ve made significant strides and pushed the boundaries of science, bringing the technology close to commercial success. However, we still face the challenge of proving its viability on a larger scale. The next decade will be crucial in determining whether we can scale up perovskite production to hundreds of gigawatts and whether the technology will be stable and impactful over a 25-year lifespan. We must demonstrate not just scientific success but real-world impact; this is both stressful and exciting, given the potential for success.

On a personal level, balancing work and family life brings its own set of challenges. As a parent of three children, I share the common concerns of whether they are doing well in school, making the right decisions about university, and navigating their social lives. These everyday worries are part of life.

To maintain a positive outlook, it’s essential not to take oneself too seriously. While it’s important to work hard and strive for success, finding a balance is crucial. As a father and husband, I can’t dedicate every waking hour to work without neglecting my family. It’s important to set boundaries and ensure that there is time to recharge. In fact, having a family can be a good reminder not to overwork, as it provides a necessary balance and helps keep work in perspective. I believe that a well-rounded life outside of work contributes positively to my effectiveness and creativity as a researcher.

Maintaining a happy mood under intense research work also requires a supportive environment, both personally and professionally. Collaboration with colleagues, celebrating small victories, and taking breaks when needed are all part of it. It is also important to have hobbies and interests outside of research to maintain a sense of balance and well-being.

**Q15. Do you know Light: Science and Application and the Light People Section on this journal? What would be your suggestion/advice to this journal and the section?**

I must be honest. I was not aware of the journal before you reached out to me to do this interview. The content seems to be highly relevant to the area I work in. Looking at the sort of papers published they seem to be mostly on spectroscopy techniques and demonstration of optical phenomena. Possibly its readership could be broadened if there were more optoelectronic device-based studies published. But this may not be the aim of the journal.

